# Oxygen-Plasma-Treated Al/TaO_X_/Al Resistive Memory for Enhanced Synaptic Characteristics

**DOI:** 10.3390/biomimetics9090578

**Published:** 2024-09-23

**Authors:** Gyeongpyo Kim, Seoyoung Park, Minsuk Koo, Sungjun Kim

**Affiliations:** 1 Division of Electronics and Electrical Engineering, Dongguk University, Seoul 04620, Republic of Korea; 2 Department of AI Semiconductor, School of Advanced Cross-Disciplinary Studies, University of Seoul, Seoul 02504, Republic of Korea

**Keywords:** resistive switching, plasma treatment, neuromorphic system, artificial synapse

## Abstract

In this study, we investigate the impact of O_2_ plasma treatment on the performance of Al/TaO_X_/Al-based resistive random-access memory (RRAM) devices, focusing on applications in neuromorphic systems. Comparative analysis using scanning electron microscopy and X-ray photoelectron spectroscopy confirmed the differences in chemical composition between O_2_-plasma-treated and untreated RRAM cells. Direct-current measurements showed that O_2_-plasma-treated RRAM cells exhibited significant improvements over untreated RRAM cells, including higher on/off ratios, improved uniformity and distribution, longer retention times, and enhanced durability. The conduction mechanism is investigated by current–voltage (I–V) curve fitting. In addition, paired-pulse facilitation (PPF) is observed using partial short-term memory. Furthermore, 3- and 4-bit weight tuning with auto-pulse-tuning algorithms was achieved to improve the controllability of the synapse weight for the neuromorphic system, maintaining retention times exceeding 10^3^ s in the multiple states. Neuromorphic simulation with an MNIST dataset is conducted to evaluate the synaptic device.

## 1. Introduction

Traditional semiconductor technologies are nearing their growth limits [1]. Moreover, the increasing speed disparity between the CPU and memory in von Neumann architecture has become a significant challenge. This problem is exacerbated by the growing volume of data. The adoption of systems that mimic the human brain represents a widely explored solution to this challenge. Neuromorphic systems emulate the organizational and operational characteristics of the biological brain, enabling efficient information processing among neurons and synapses. Because of parallel processing, these interconnected neurons and synapses facilitate rapid information transfer, resulting in high energy efficiency [2]. Variability in signal transmission, influenced by the activity of presynaptic and postsynaptic neurons, leads to changes in synaptic strength. This process, known as synaptic plasticity, is the ability of the brain to learn and retain memory [3]. Synaptic plasticity is generally classified as short-term memory (STM) and long-term memory (LTM). Short-term plasticity is crucial for quick responses and immediate information processing, lasting from a few milliseconds to several minutes. By contrast, long-term plasticity, which is important for sustained memory retention, can extend from several hours to days [4]. To harness these advantages, neuromorphic computing systems have been developed [5,6], incorporating various types of resistance-based memories such as ferroelectric random-access memory [7,8], phase-change RAM [9], magnetic RAM [10], and resistive RAM (RRAM) [11]. Among them, RRAM is particularly notable for its straightforward two-terminal design, rapid operation, low energy consumption, high endurance, extended retention times, and compatibility with CMOS manufacturing processes. By applying a voltage bias across the electrodes, the device can be switched between a high-resistance state (HRS) and a low-resistance state (LRS), making these states ideal for data storage.

A typical RRAM device comprises a resistive-switching (RS) layer, flanked by two metal electrodes, mimicking the configuration of a simple two-terminal capacitor. Over the past two decades, a broad spectrum of materials, including metal oxides such as Ta_2_O_5_, HfO_2_, ZrO_2_, TiO_2_, ZnO, Al_2_O_3_, and CeO_2_, organic compounds [12], newly developed perovskites [13], and two-dimensional materials [14], have been explored for RS memory in neuromorphic applications [15,16,17,18]. Because of their outstanding compatibility with semiconductor manufacturing technologies, cost-effectiveness, multistate switching capability, high switching speeds, reliability, compact cell sizes, and low power consumption, oxide-based materials have garnered significant interest in RS memory fabrication [19]. During the initial forming process, conductive oxygen vacancy filaments are created within the oxide layer, linking the two electrodes and inducing an LRS when a relatively high voltage is applied to the memristor device. Numerous studies have identified the formation of oxygen vacancies at the oxide/ohmic electrode interface during processing and subsequent forming steps [20]. However, the potential for large-scale commercial deployment of RRAM devices is often undermined by the unpredictable nature of conductive filament (CF) formation in oxide-based memories during switching operations. Endurance, repeatability, and uniformity during the switching cycles of these memory devices continue to pose significant challenges, impeding advancements in this technology. To mitigate this issue, several effective strategies have been developed, including different process optimization techniques, such as metal doping, integration of reactive metal layers, development of multilayer architectures, and incorporation of metal nanocrystals, aimed at addressing the variability challenges associated with RRAM memory devices [21,22,23,24]. To enhance the endurance characteristics of RRAM devices, various research efforts have been undertaken, including the intercalation of additional materials [25,26], doping with other elements [27,28,29], and controlling annealing conditions [30]. These modifications are aimed at optimizing device performance by improving the stability and durability of a device during repeated switching cycles.

Among the various studies focused on improving the switching characteristics of RRAM devices, oxygen plasma treatment (OPT) has attracted attention as an effective method for controlling the concentration of oxygen vacancies and enhancing the quality of the switching layer [31,32,33,34,35,36,37]. This technique helps optimize device performance by ensuring more uniform and reliable switching characteristics. In this study, we investigated the effects of OPT on enhancing the durability and stability of TaO_X_-based RRAM devices. The TaO_X_ switching layer was deposited at a thickness of 40 nm using a sputtering system. For comparative analysis, two types of TaO_X_-based resistive memory devices were fabricated: the first device without OPT (Al/TaO_X_/Al) and the second device with OPT applied to both the deposited TaO_X_ layer and the bottom electrode (Al/TaO_X_ (OPT)/Al (OPT)). The elemental composition of the TaO_X_ layers, both with and without OPT, was investigated by X-ray photoelectron spectroscopy (XPS). The Al/TaO_X_ (OPT)/Al (OPT) device exhibited typical bipolar RS behavior, achieving a retention time exceeding 10^3^ s, an on/off ratio greater than 10, and improved LRS and HRS distribution by OPT. Additionally, current–voltage (I–V) curves were analyzed to further investigate the resistance-switching mechanisms within the device. Multistep functionality was demonstrated in the Al/TaO_X_ (OPT)/Al (OPT) device by adjusting the current compliance (CC), with a long dwell time (10^3^ s) observed at each step. This device also demonstrated the ability to reproduce several synaptic properties, such as paired-pulse facilitation (PPF) and capabilities pertinent to reservoir computing.

## 2. Materials and Methods

A SiO_2_ substrate was used to prepare the device. First, a 100 nm thick layer of Al was deposited as the bottom electrode by DC sputtering. Then, a 40 nm thick layer of TaO_X_ was deposited as insulation on the bottom electrode by radio frequency (RF) sputtering. Finally, a 100 nm thick Al layer was deposited as the top electrode by DC sputtering. The substrate was maintained at room temperature during TaO_X_ deposition, and the working pressure was 3 mTorr. Ar and O_2_ gases flowed over the substrate at 20 and 6 sccm, respectively, while applying an RF power of 240 W. Further, patterning of the Al top electrode was achieved using the lift-off method. O_2_ plasma treatment was performed for 5 min at an RF power of 100 W in an environment of 100 mTorr and 50 sccm O_2_. The detailed fabrication scheme is presented in S1. As described in Figure S1, the fabrication process of the Al/TaO_X_ (OPT)/Al (OPT) device begins with OPT of the bottom Al electrode. Following this, a TaO_X_ layer was deposited on the plasma-treated Al surface, and the structure underwent another round of oxygen plasma treatment. Through the OPT process, the structure and electrical properties of TaO_X_ are improved, enabling it to function effectively as the active layer. Oxygen plasma treatment reduces defects in the TaO_X_, enhances material density and uniformity, and strengthens its insulating properties. Finally, the top Al electrode was deposited, followed by a lithography. Etching processes were then conducted to complete the fabrication of the Al/TaO_X_ (OPT)/Al (OPT) device. The cross-section of the Al/TaO_X_/Al device was observed using scanning electron microscopy (SEM), and the physical properties were analyzed by XPS. The device transmittance was measured using a spectrophotometer, and the I–V as well as transient curves of the RRAM cells were measured using a semiconductor parameter analyzer (Keithley 4200-SCS and 4225-PMU ultrafast module) while applying a bias to the top electrode and grounding the bottom electrode.

## 3. Results and Discussion

**Figure 1:**
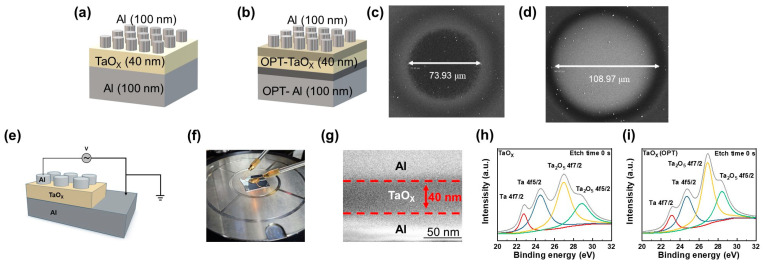
Schematic diagram of the two fabricated Al/TaO_X_/Al devices: (**a**) without OPT and (**b**) with OPT. Images of the top electrodes for (**c**) Al/TaO_X_/Al and (**d**) Al/TaO_X_ (OPT)/TaO_X_ (OPT) structures. (**e**) Current injection diagram. (**f**) Actual measurement image. (**g**) SEM image of an Al/TaO_X_/Al device. XPS spectra of Ta 4f on the (**h**) TaO_X_ layer and (**i**) TaO_X_ layer with OPT.

Figure 1a,b depict the configurations of the Al/TaO_X_/Al and Al/TaO_X_ (OPT)/Al (OPT) devices, respectively. For both devices, a 100 nm Al layer serves as the bottom electrode, on top of which a 40 nm TaO_X_ layer is deposited. The top electrode, patterned and aligned over the TaO_X_ layer, is also composed of 100 nm thick Al. The top electrode diameter of the Al/TaO_X_/Al device is 73.93 μm (Figure 1c), while that of the Al/TaO_X_ (OPT)/Al (OPT) device is 108.97 μm (Figure 1d). The measurement voltage conditions of the devices are shown in Figure 1e. Electrical measurements were conducted by applying a voltage to the top electrode. The actual measurement image is shown in Figure 1f. Figure 1g presents an SEM image confirming the thickness of the insulating layer as 40 nm and its composition within a metal–insulator–metal (MIM) structure. The effect of OPT on the device is illustrated in Figure 1h,i. The composition and chemical bonding state of the TaO_X_ layer interface was analyzed using XPS; the corresponding spectra are displayed in Figure 1d,e. The peaks at 22.33, 23.96, 26.5, and 28.4 eV correspond to Ta 4f7/2 (yellow), Ta 4f5/2 (blue), Ta_2_O_5_ 4f7/2 (red), and Ta_2_O_5_ 4f5/2 (green), respectively [38,39,40,41]. In the TaO_X_ (OPT) device, the Ta_2_O_5_ peak at the interface is more pronounced than the Ta peak compared to the device without OPT, indicating that OPT enhances the oxidation of Ta at the interface. This modification possibly contributes to a reduction in oxygen vacancies [42]. To assess the stoichiometry changes in the TaO_X_ layer, we extracted the O 1s spectra from XPS analysis and compared the oxygen vacancies in the layer. For the Al/TaO_X_/Al device, the binding energy corresponding to oxygen vacancies was found to be 528.3 eV, which is relatively high. In contrast, for the Al/TaO_X_ (OPT)/Al (OPT) device, the binding energy of the oxygen vacancies was 520.63 eV, indicating a lower oxygen vacancy concentration compared to the untreated device. These results suggest that oxygen plasma treatment effectively reduces oxygen vacancies. However, this alteration is confined to the interface, as evidenced by Figure S2a,b, which show no significant difference in the ratio of the Ta_2_O_5_ peak to the Ta peak between the devices. This suggests that their composition and chemical bonding state remain similar beyond the interface. Furthermore, the effect of OPT on the bottom electrode (Al) is shown in Figure S3a–d. A higher oxygen ratio is detected at the interface of the device with OPT applied to the Al layer compared to the untreated device. However, this effect is localized at the interface and does not extend deeper into the device structure.

**Figure 2:**
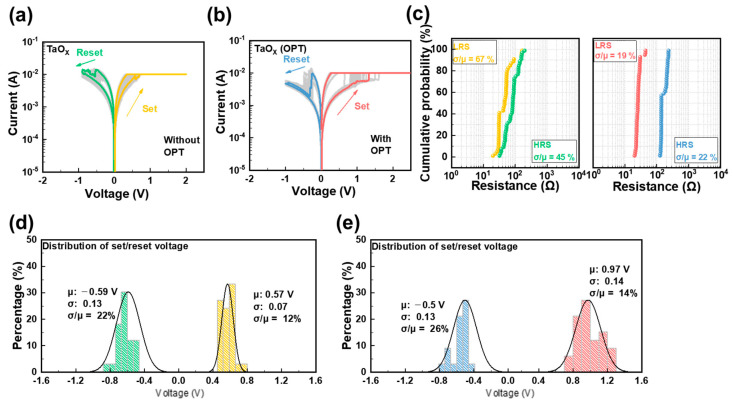
I–V characteristics of each RRAM device for 30 cycles: (**a**) Al/TaO_X_/Al device and (**b**) Al/TaO_X_ (OPT)/Al (OPT) device. (**c**) Distribution characteristics of HRS/LRS for 50 cells on each device. (**d**) Distribution characteristics of the set/reset voltage for the Al/TaO_X_/Al device. (**e**) Distribution characteristics of the reset voltage for the Al/TaO_X_ (OPT)/Al (OPT) device.

Typical I–V curves for both devices are presented in Figure 2a,b. These curves were recorded using a DC dual sweep and exhibited the classical bipolar resistance-switching characteristics, alternating between HRS and LRS [43]. To prevent permanent damage to the switching film from overflow current, the CC was limited to less than 10 mA. Like typical RRAM devices, each device required a forming process; the I–V curves from this process are shown in Figure S4a,b [44]. While there have been improvements in the performance of RRAM devices, the on/off ratio remains relatively low compared to other studies in the literature. To tackle this issue, several strategies are being explored to enhance the overall performance. Firstly, optimizing the composition and thickness of the active layer could significantly influence the on/off ratio by enabling more precise control over carrier concentration and mobility. Changes in the active material can lead to better-switching characteristics, which would improve device efficiency. Secondly, enhancements in the electrode material and structure may help lower contact resistance, thereby boosting the overall electrical performance of the RRAM device. Reducing contact resistance can facilitate better current flow and switching behavior, both essential for achieving higher on/off ratios. Thirdly, careful optimization of fabrication parameters, such as deposition temperature and pressure, is expected to affect defect density. By controlling these parameters, it may be possible to minimize defects in the active layer, which can enhance electrical characteristics and the overall stability of the device. Lastly, post-fabrication processes like annealing could further improve material crystallinity and reduce defects, positively impacting the on/off ratio. Annealing has been shown to enhance the structural properties of materials, leading to more stable and efficient switching behavior. Future research will focus on exploring these approaches in greater detail to achieve more competitive device performance and higher on/off ratios. For an accurate comparison of the switching characteristics of the two fabricated devices, Figure 2c displays the statistical distributions of LRS and HRS to assess device uniformity at a read voltage of 0.1 V. After evaluating the LRS and HRS distributions over more than 50 cycles in both Al/TaO_X_/Al and Al/TaO_X_ (OPT)/Al (OPT), it was confirmed that the device with OPT exhibited a more uniform distribution of LRS and HRS. Uniformity was assessed using the coefficient of variation, with the coefficients of variation for LRS and HRS in Al/TaO_X_/Al being 67% and 45%, respectively. By contrast, the coefficients of variation for LRS and HRS in Al/TaO_X_ (OPT)/Al (OPT) were lower, at 19% and 22%, respectively. Further, as illustrated in Figure 2d,e, a 10-cycle DC sweep was conducted on each of five randomly selected cells to assess the set and reset voltage distributions. The results indicated that the set voltage for the Al/TaO_X_ (OPT)/Al (OPT) device, which underwent OPT, required a higher voltage compared to the Al/TaO_X_/Al device. However, the reset voltage exhibited no significant difference between the devices. Additionally, the coefficients of variation for the set/reset voltages were similar across both devices, indicating that the uniformities of the voltage required for state transitions during repeated set/reset cycles were similar. 

**Figure 3:**
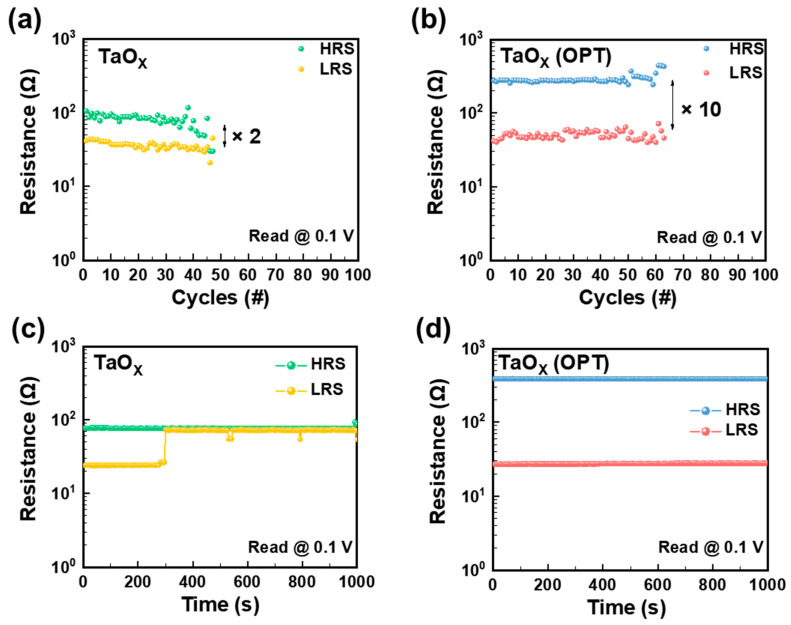
Endurance of the (**a**) Al/TaO_X_/Al device and (**b**) Al/TaO_X_ (OPT)/Al (OPT) device measured by repetitive DC voltage application. Retention measurement for the (**c**) Al/TaO_X_/Al device and (**d**) Al/TaO_X_ (OPT)/Al (OPT) device at a constant voltage of 0.1 V.

Figure 3 illustrates the retention and endurance characteristics of both devices. Figure 3a,b demonstrate that the durability and on/off ratio of devices that underwent OPT are significantly improved compared to those of the devices without OPT. Specifically, the Al/TaO_X_/Al device without OPT showed a decrease in the resistance of the HRS after approximately 40 cycles, at which point the cycle could not be repeated any further. During these cycles, the average on/off ratio was only 2. Conversely, the Al/TaO_X_ (OPT)/Al (OPT) device, which underwent OPT, exhibited enhanced durability, for approximately 60 cycles, and an improved on/off ratio of 10. Furthermore, Figure 3c,d reveal that the on/off ratio has been enhanced by OPT. In addition, Figure 3c,d present the retention time of each device. For the Al/TaO_X_/Al device, there was an increase in resistance after maintaining the LRS for approximately 300 s. By contrast, the Al/TaO_X_ (OPT)/Al (OPT) device could stably maintain each state for more than 10^3^ s, evidencing the positive impact of OPT on retention time. These results indicate that OPT processing offers several advantages, such as improved on/off ratio, switching uniformity, retention, and endurance. However, the operating voltage increases, a detail that can be numerically verified in Table 1.

**Figure 4:**
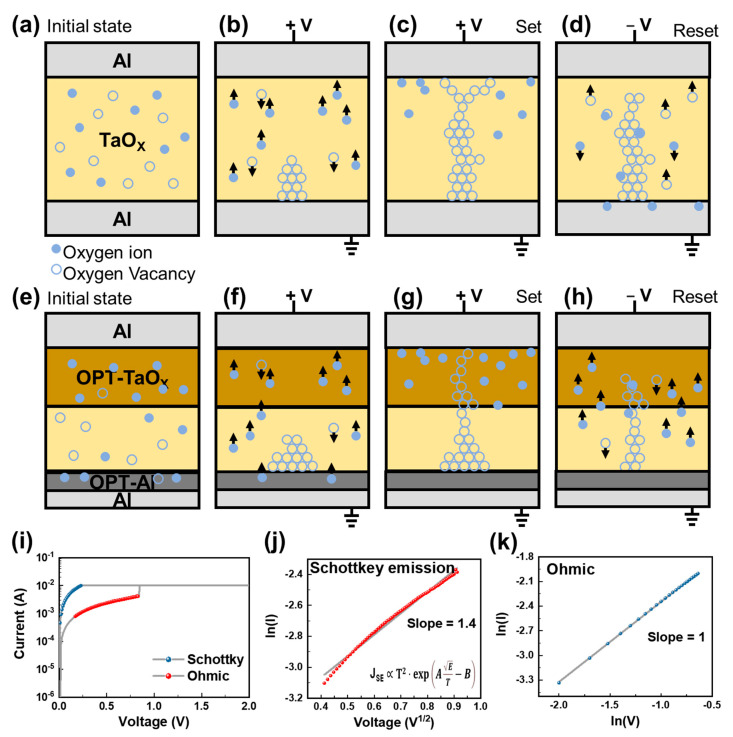
Schematic diagram of the switching mechanism. The Al/TaO_X_/Al device is in the (**a**) pristine state, (**b**) with a positive voltage, (**c**) LRS, and (**d**) with a negative voltage. The pristine state of the Al/TaO_X_ (OPT)/Al (OPT) device. Continuous application of a positive voltage. The Al/TaO_X_ (OPT)/Al (OPT) device in the (**e**) pristine state, (**f**) with a positive voltage, (**g**) LRS, and (**h**) with a negative voltage. (**i**) Typical I–V curve of the OPT. (**j**) ln(I) versus V1/2 plot for the Schottky emission mechanism (red). (**k**) Linear I–V plot in the log–log scale for ohmic conduction (blue).

The schematic diagrams in Figure 4a–d detail the switching mechanism of the Al/TaO_X_/Al device. Figure 4a displays the initial state of the device. Upon the application of a positive (+) SET voltage to the top electrode, oxygen ions migrated toward the top electrode, while oxygen vacancies moved toward the bottom electrode owing to the internal electric field, leading to the formation of CFs, as depicted in Figure 4b. With the continued application of a positive voltage, these CFs—primarily composed of oxygen vacancies—bridged the top and bottom electrodes, transitioning the device into an LRS, as illustrated in Figure 4c. Conversely, when a negative (−) RESET voltage was applied to the top electrode, oxygen ions recombined with the oxygen vacancies, disrupting the CFs, as shown in Figure 4d. With repeated switching cycles, the concentration of oxygen ions diminishes because of the oxygen-absorbing characteristics of the Al bottom electrode [45]. This reduction in oxygen ions interrupts the RESET process and degrades the switching capability of the device. The schematic diagrams in Figure 4e–h illustrate the switching mechanism of the Al/TaO_X_ (OPT)/Al (OPT) device. XPS data revealed that the OPT-treated TaO_X_ layer showed varying ratios of bonded oxygen-to-oxygen vacancies depending on the location within the layer. Specifically, the upper TaO_X_ layer had more oxygen bonded to tantalum, indicating a reduction in oxygen vacancies. By contrast, the lower TaO_X_ layer showed minimal changes compared to its state before OPT treatment. Oxygen vacancies are crucial for forming CFs within the TaO_X_ layer. Initially, as shown in Figure 4e, the TaO_X_ (OPT) layer contains more oxygen ions compared to the untreated TaO_X_ layer. When a positive SET voltage is applied to the top electrode, oxygen ions move toward the top electrode, while oxygen vacancies migrate toward the bottom electrode because of the electric field, forming CFs, as depicted in Figure 4f. A higher content of oxygen vacancies within the layer leads to the formation of thicker filaments, as explained by the CF filament model shown in Figure 4g [46]. The filament ruptures at its weakest part owing to localized Joule heating, which occurs in the TaO_X_ (OPT) layer, switching the device from an LRS to an HRS, as indicated in Figure 4h. The TaO_X_ (OPT) layer helps stabilize the formation and rupture of CFs in the TaO_X_-based device, resulting in a more stable switching performance [46]. Additionally, thinner CFs lead to a decrease in resistance due to the reduction in cross-sectional area [47]. Therefore, the HRS of the Al/TaO_X_ (OPT)/Al (OPT) device exhibits a higher resistance, thus increasing the on/off ratio. Unlike the Al/TaO_X_/Al device, the Al/TaO_X_ (OPT)/Al (OPT) device does not experience a reduction in oxygen ion content under repeated switching cycles. This is due to the oxygen-absorbing characteristics of the Al bottom electrode. The presence of more oxygen at the Al bottom electrode, resulting from the OPT treatment, facilitates more stable cycling, ensuring the retention of oxygen ions and thereby maintaining stable switching characteristics over time.

To understand the physics behind the resistance-switching characteristics of the Al/TaO_X_ (OPT)/Al (OPT) device, we employed extensively studied methods for identifying conduction mechanisms from I–V characteristics [48]. Figure 4i–k illustrate the conduction mechanism analysis via I–V curve fitting, confirming that the switching involves a combination of Schottky emission and ohmic conduction. Figure 4j presents the ln(I) vs. V1/2 plot for the HRS, which is indicative of Schottky emission. In the LRS, ohmic conduction is identified, where electrons could move through the CF. Figure 4k shows the linear I–V plot confirming the ohmic conduction mechanism under an applied bias. 

**Figure 5:**
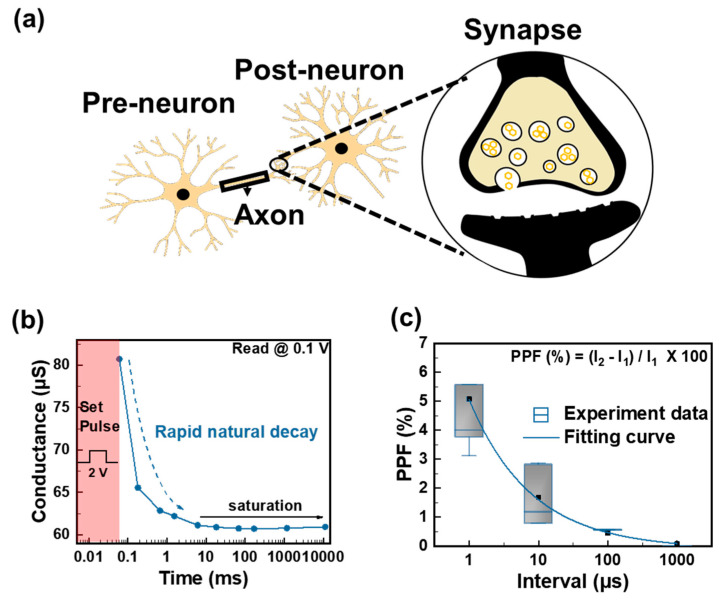
(**a**) Biological system and schematic diagram of a memristor device system used as an artificial synapse. (**b**) Natural decay process involving a set pulse in the interval times between the pulses. The trend line (Rapid natural decay) and solid line of the decay (saturation). (**c**) Statistical distribution of PPF as a function of the interval time. (Experiment data and Fitting curve).

As illustrated in Figure 5a, biological synapses function to transmit electrical or chemical signals between two neurons, comprising presynaptic and postsynaptic components. The presynaptic component is triggered to release neurotransmitters by an action potential, which is also known as a nerve impulse. In biological synapses, when an action potential is received, Ca^2+^ or K^+^ influx occurs in the synaptic cleft, adjusting the synaptic weight between the presynaptic and postsynaptic neurons [49]. TaO_X_-based RRAM devices mimic the shape and function of biological synapses and are activated by the migration of oxygen vacancies (neurotransmitters) induced by positive pulses (action potentials) acting as presynaptic inputs. These oxygen vacancies move to the bottom electrode, which acts as the postsynaptic component, and form CFs. As shown in Figure 5b, the partial rapid-natural-decay characteristics of the Al/TaO_X_ (OPT)/Al (OPT) device are utilized to measure PPF. After applying the set pulse (amplitude: 3 V; width: 50 μs), read pulses (amplitude: 0.1 V; width: 50 μs) were applied at increasing intervals. The intervals of the applied read pulses, ranging from 100 μs to 10 s, are shown in Figure S6a. Decay occurred in approximately 1 ms before becoming saturated and sustained, indicating partial STM characteristics. This result contrasts with the retention exceeding 10^3^ s in the Al/TaO_X_ (OPT)/Al (OPT) device, as depicted in Figure 3d As seen in Figure S5a, a small window appears even when the set process is repeated without the reset process after conducting the set process in a DC sweep, indicating the occurrence of partial STM. Figure S5b shows the result of continuous application of a read voltage without intervals after the set pulse (amplitude: 2 V; width: 50 μs). The conductance increased sharply after the set pulse and then decayed, further supporting the occurrence of the partial STM. As depicted in Figure 5c, PPF was measured using the partial STM characteristics of the Al/TaO_X_ (OPT)/Al (OPT) device. PPF is a well-known type of short-term synaptic plasticity in biological synapses that plays a crucial role in temporal information processing [50,51]. Specifically, the operation of PPF is based on successive time intervals between pre- and post-pulses. As the time interval between pre- and post-pulses decreases, the postsynaptic weight increases. This is demonstrated using paired pulses (amplitude: 3 V; width: 5 μs) at different interval times (1 μs, 10 μs, 100 μs, and 1 ms). The average current of the pre-pulse is lower than that of the post-pulse at all interval times. When a short stimulation period is applied to biological synapses, a shorter interval time results in a higher change rate in the second pulse compared to the first pulse, like having stable memory. Conversely, when a long stimulation period is applied to biological synapses, a longer interval time results in a lower change rate in current, indicating an unstable memory [52]. Therefore, PPF can be used to evaluate the extent of the synaptic weight. The pulse scheme used for PPF measurement is shown in Figure S6b.

**Figure 6:**
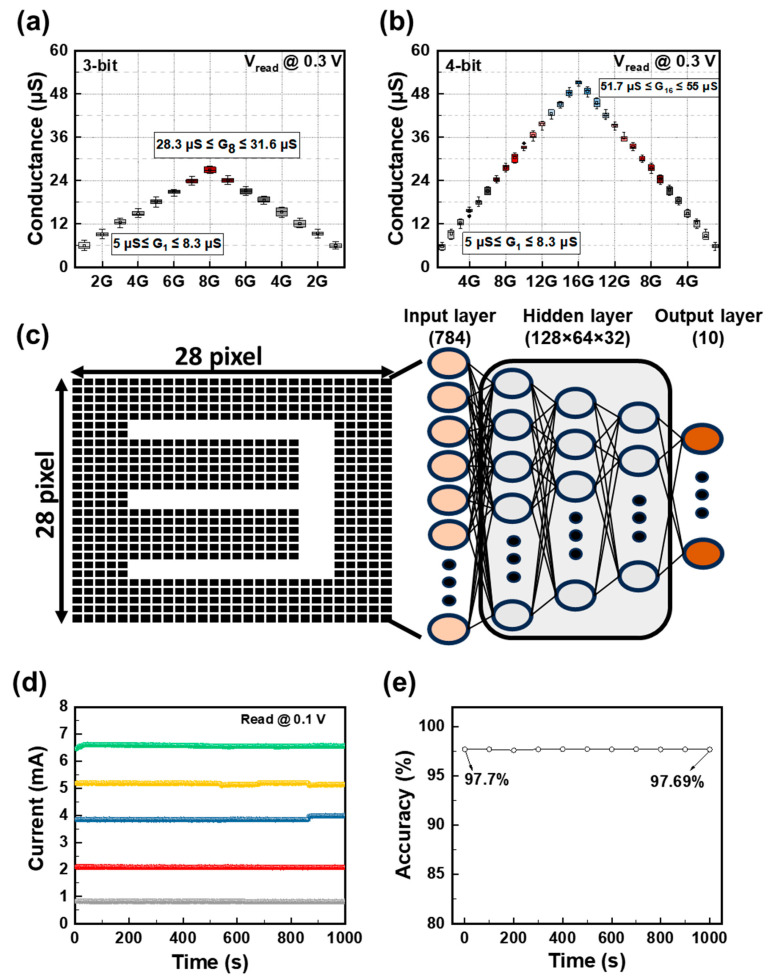
Auto-conductance-tuning method for the MLC implementation, with box charts used to represent each level of the (**a**) 3-bit MLC (G_1_ ranges from 5 μs to 8.3 μs, and G_8_ ranges from 28.3 μs to 31.6 μs) and (**b**) 4-bit MLC (G_1_ ranges from 5 μs to 8.3 μs, and G_16_ ranges from 51.7 μs to 55 μs). (**c**) MNIST classification accuracy. (**d**) Multilevel retention measurement with different CCs (1 mA (grey), 3 mA (red), 5 mA (blue), 7 mA (yellow), 10 mA (green)). Retention test for up to 103 s in multiple states. (**e**) Recognition accuracy of the trained retention system on the MNIST dataset.

The neuromorphic system performances of the Al/TaO_X_ (OPT)/Al (OPT) device in multilevel operation are presented in Figure 6. Multilevel conductance (MLC) has the potential to store data in digital storage devices and retain synaptic weights in neuromorphic systems. To improve inference accuracy in biological neural networks, RRAM devices capable of updating synaptic weights with three bits or more are required, and accuracy can be enhanced by increasing the capacity of synaptic weights within a single RRAM device [53]. Despite the potential for increased current variation due to randomly distributed oxygen vacancies within the TaO_X_ (OPT) switching layer, the Al/TaO_X_ (OPT)/Al (OPT) device can achieve multiple conductance levels through the auto-weight-tunning technique to regulate set and reset amplitudes [54]. However, adjusting to the desired target conductance involves a trade-off with increasing pulse repetition cycles. The read pulses (0.3 V/100 μs) are utilized after set and reset pulses to verify whether the current state of the device has reached the target current tolerance range. Set pulses are applied if the read pulse falls below the target tolerance range, while reset pulses are applied if the read pulse exceeds the target tolerance range. Set and reset pulses with a pulse width of 100 μs progressively increased and decreased from 0 to ±2 V in increments of ±25 mV to facilitate target tuning. Additionally, the maximum and minimum values of the target current were set at 5 and 55 μA, respectively. Figure S7 demonstrates the pulse endurance characteristics for 3- and 4-bit weight tuning. During 10 cycles of programming and erasing, precise tuning of target weights could be achieved for both 3- and 4-bit MLC. The tuning results for 3- and 4-bit MLC are depicted in the box charts in Figure 6a,b, confirming successful tuning without any overlap between each pair of states. To verify the inference accuracy using the offline-learning-based neural network of the Al/TaO_X_ (OPT)/Al (OPT) device, a neural network with three hidden layers was configured to evaluate the classification performance on a Modified National Institute of Standards and Technology (MNIST) dataset under retention loss, as shown in Figure 6c [55]. The simulation incorporated weight changes due to retention loss in each fully connected layer. To reflect the retention loss characteristics of the neural network, the changes in accuracy according to retention must be determined using trained weights. Therefore, an offline-learning-based neural network was used to verify the accuracy during inference situations. After training the artificial neural network for over 10 epochs, weight quantization was set to five bits. A cross-entropy loss function and the Adam optimizer were used to determine the weights. The Al/TaO_X_ (OPT)/Al (OPT) device used in the simulation showed various retention times, confirming that there was no data loss for over 10^3^ s, as shown in Figure 6d. Consequently, in the simulation, the maximum weight loss for 10^3^ s was close to 0%, resulting in no degradation in recognition accuracy over time. In Figure S8a, the DC I–V curves of the D2 device under various compliance current (CC) conditions (1 mA, 3 mA, 5 mA, 7 mA, and 10 mA) are presented. The classification accuracy obtained using the MNIST dataset, based on the programmed current range, is shown in Figure 6e. The accuracy loss was only 0.01%, indicating that data retention and recognition rates were maintained over long periods. In conclusion, the Al/TaO_X_ (OPT)/Al (OPT) device, characterized by excellent data retention properties, abrupt RS, and a memory window exceeding 10^3^ in various states, is suitable as a synaptic memristor for offline learning. Additionally, the ability to achieve MLC characteristics of four bits or more by the auto-weight-tunning method can enhance inference accuracy in neuromorphic systems and demonstrate high MNIST recognition rates without accuracy loss. 

**Figure 7:**
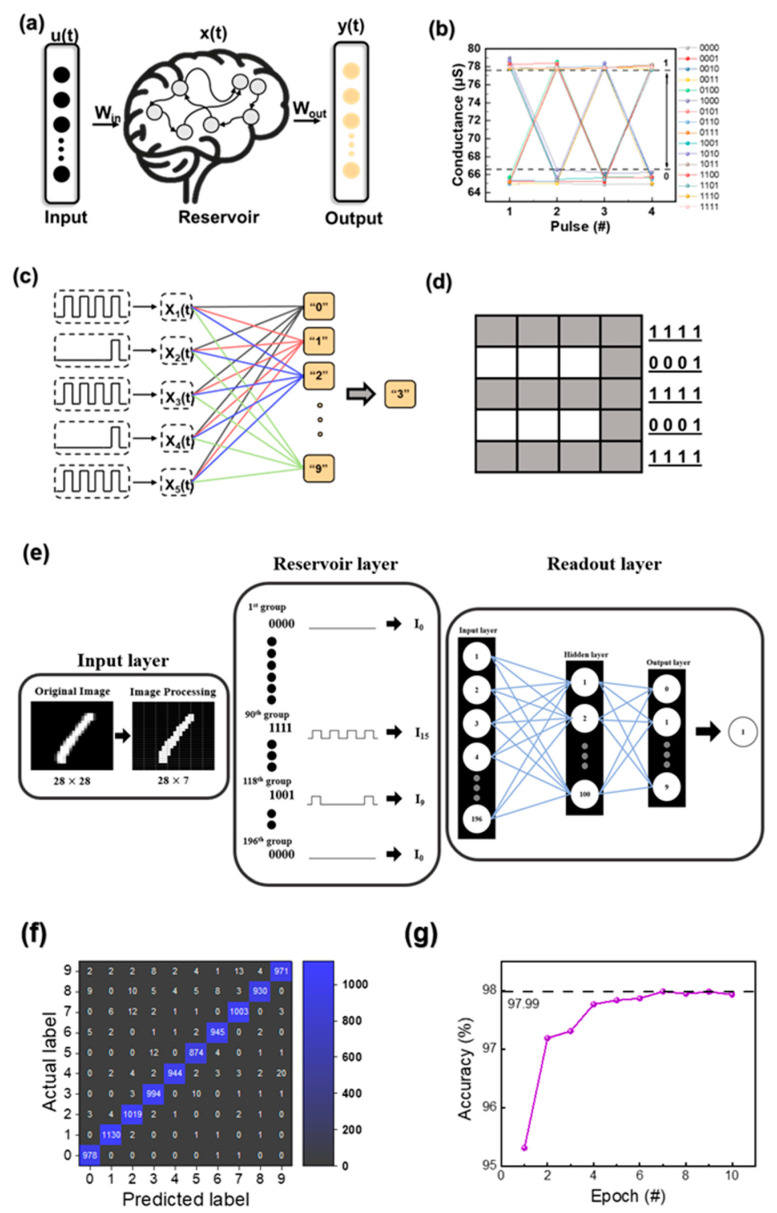
(**a**) Concept of reservoir computing. (**b**) Sixteen states as per specific pulse streams. (**c**) Reservoir computing system with a pulse train, RRAM devices, and output node. (**d**) Digit “3” implemented using 5 × 4 pixels. (**e**) Conceptual diagram of reservoir computing with pixel binarization. (**f**) Training of the readout layer using a CNN on the MNIST dataset. (**g**) Recognition accuracy of the trained reservoir computing system on the MNIST dataset.

Finally, the partial STM characteristics of the Al/TaO_X_ (OPT)/Al (OPT) device were utilized to develop a reservoir computing system. Reservoir computing introduces a new dimension of nonlinear transformation of input signals through a space called the reservoir, developed in recurrent neural networks. This can be applied to pattern recognition, speech recognition, and time series prediction [56]. RRAM devices exhibit different conductance states over time depending on the shape of the applied input signals. A memristor with partial STM characteristics has the advantage of being able to accumulate multiple states in a single memristor cell by adjusting the pulse intervals of the applied voltage set. The role of the input layer is to recognize the timing of the input, and the output is connected to a high-dimensional computational space. The reservoir layer provides sufficient dynamic data to map the input to a high-dimensional output. By applying four consecutive pulse streams, 4-bit data can be stored temporarily in a single memristor. The time intervals are adjusted to represent 0 and 1. Figure 7a shows the system composed of three components: a single input layer, a dynamic reservoir layer, and an output layer. The temporal input signal u(t) is applied to the reservoir, which comprises multiple nodes. The input signal is then mapped to a new high-dimensional space represented by x(t), the collective state of all neurons in the reservoir, and nonlinear transformation is performed. The obtained reservoir state x(t) is analyzed using the readout function. Finally, the output y(t) is derived linearly by learning via readout. Figure 7b shows the representation of a total of 16 states from 0000 to 1111 in a 4-bit format by the Al/TaO_X_ (OPT)/Al (OPT) device with STM characteristics. In all states, “1” has a conductance value greater than 77 μS, and “0” has a conductance value less than 67 μS. Therefore, the difference between “1” and “0” is distinguishable. Data representing a total of 16 states from 0000 to 1111 in a 4-bit format are shown in Figure S9. Figure 7c illustrates a schematic of the pulse input layer, reservoir layer, and readout through pulse streams. After the four input pulse streams process the temporal features of the reservoir, the goal is to extract the digit 3 as an image. Five RRAMs are used to recognize the image, with each memristor processing the input pulse stream into a specific row of the image. When four input pulse streams are input as 4-bit pulses, one for each of the five lines, the STM effect of the device changes state. Then, with a simple learning algorithm, 4-bit data are temporarily stored in a single RRAM. Figure 7d shows the character image “3” composed of 5 × 4 pixels stored using the readout function. Binary data represent “1” as black pixels and “0” as white pixels. With the four pulses, each row can be represented by 1s and 0s. The number 3 is represented in binary using [1111] for rows 1, 3, and 5 and [0001] for rows 2 and 4. Figure 7e illustrates the concept of reservoir computing. In the input layer, the brightness information of each pixel from the original image is binarized, where values from 0 to 127 are assigned a value of 0, and values from 128 to 255 are assigned a value of 1. Subsequently, every four pixels are grouped, reducing the 28 × 28 image to 7 × 28, resulting in a total of 196 groups. Each group represents one of the 16 possible states, ranging from 0000 to 1111. When transitioning from the input layer to the reservoir layer, each group is associated with one of these 16 states, which is then mapped to a corresponding current value measured in the physical reservoir. This process is equivalent to generating 196 input neurons in the readout layer. The current values obtained from the reservoir computing process are scaled between 0 and 1 using min–max normalization, and these normalized values are then fed into the input layer of the readout layer. The values are processed through a hidden layer, and the final outcome is produced in the output layer. Figure 7f demonstrates how the readout layer is trained using a convolutional neural network (CNN) to tackle complex problems, specifically evaluated using the MNIST image recognition database. As shown in Figure 7g, the recognition rate is notably high.

## 4. Conclusions

The application of O_2_ plasma treatment to Al/TaO_X_/Al RRAM devices significantly enhances their performance, making them highly suitable for neuromorphic applications. Our comparative analysis using SEM and XPS confirmed the chemical composition changes induced by O_2_ plasma treatment. The treated RRAM exhibited superior electrical characteristics, including a higher on/off ratio, better uniformity and distribution, extended retention time, and improved endurance. Confirmation of the operating mechanism by I–V curve fitting and the observed partial STM effect facilitated the demonstration of PPF. The 3- and 4-bit implementation with an auto-conductance-tuning algorithm demonstrated the device’s precise multilevel control capability. MNIST simulation results highlight the long-term stability of recognition rates, and the successful demonstration of reservoir computing underscores the potential of O_2_-plasma-treated RRAM in neuromorphic systems. These findings pave the way for further advancements in RRAM technology, offering promising prospects for future neuromorphic applications. In our study, we applied a specific oxygen plasma treatment to the RRAM devices to optimize their performance. The selected plasma treatment parameters were based on preliminary optimization studies aimed at achieving a balance between forming an adequate oxide layer and minimizing damage to the underlying materials. Specifically, we chose a moderate plasma power and treatment time that we found to enhance the switching characteristics by improving the uniformity of the oxygen vacancies, which are crucial for resistive switching in RRAM devices. While we did not explore a wide range of plasma treatment times or power levels in this study, we recognize that these variables can significantly influence the concentration and distribution of oxygen vacancies and, consequently, the on/off ratio, endurance, and retention characteristics of the device. For instance, longer plasma treatment times could potentially lead to an excessive creation of oxygen vacancies, which may cause instability in the switching behavior. On the other hand, higher plasma power could increase the density of surface states, potentially enhancing device performance, but also increasing the risk of damage to the active layer. Given the complexity and sensitivity of RRAM devices to plasma treatment conditions, we believe that a systematic investigation into varying these parameters could yield valuable insights into optimizing device performance. Although our current study focused on a specific set of optimized conditions, we consider this an important area for future work and plan to explore these variables in greater detail in subsequent research.

## Figures and Tables

**Table 1:** Comparison of resistance-switching parameters of devices according to OPT.

Device Structure	V_SET_	V_RESET_	On/Off Ratio	Retention Time	Endurance	LRS CV	HRS CV
Al/TaO_X_/Al	0.57 V	−0.59 V	2	3 × 10^2^ s	45	67%	45%
Al/TaO_X_(OPT)/Al(OPT)	0.97 V	−0.5 V	10	10^3^ s	60	19%	22%

## Data Availability

The original contributions presented in the study are included in the article, further inquiries can be directed to the corresponding author.

## Supplementary Materials

The following supporting information can be downloaded at: https://www.mdpi.com/article/10.3390/biomimetics9090578/s1, Figure S1. (a–e) Al/TaO_X_ (OTP)/Al (OTP) device fabrication process schematic diagram; Figure S2. X-ray photoelectron spectra (XPS) of O 1s for (a) Al/TaO_X_/Al and (b) Al/TaO_X_ (OPT)/Al (OPT); Figure S3. XPS spectra of Ta 4f on the (a) TaO_X_ layer and (b) TaO_X_ layer with O_2_ plasma treatment when the etch time was 100 s. XPS depth profiles of Al and O elements in (c) Al/TaO_X_/Al device and (d) Al/TaO_X_ (OPT)/Al (OPT) device; Figure S4. Forming process of the TaO_X_ based RRAM each device (a) Al/TaO_X_/Al device and (b) Al/TaO_X_ (OPT)/Al (OPT); Figure S5. Partial short-term characteristics; Figure S6. (a) Initial set pulses and read pulse with delay. (b) Pulse scheme of paired-pulse facilitation (PPF); With a delay between each two pulses (1 μs, 10 μs, 100 μs, 1 ms); Figure S7. Multilevel switching characteristics of Al/TaO_X_ (OPT)/A l(OPT) device over 10 cycles for (a) 3-bit MLC and (b) 4-bit MLC; Figure S8. (a) DC I–V curves of the D2 with different CC conditions (1 mA, 3 mA, 5 mA, 7 mA, 10 mA); Figure S9. (a–p) Each sixteen states as per specific pulse streams.
