# Supplementary material for: Oxygen-Plasma-Treated Al/TaOX/Al Resistive Memory for Enhanced Synaptic Characteristics
Source: Biomimetics (Basel). 2024 Sep 23;9(9):578. doi: 10.3390/biomimetics9090578 (PMC11430571; doi:10.3390/biomimetics9090578)
Supplement: Supplementary file 1 [file biomimetics-09-00578-s001.zip › biomimetics-3153903-supplementary.pdf]

Article

Oxygen Plasma Treated Al/TaOX/Al Resistive Memory for Enhanced Synaptic Characteristics

Gyeongpyo Kim, Seoyoung Park, Minsuk Koo*, Sungjun Kim*

Division of Electronics and Electrical Engineering, Dongguk University, Seoul 04620, Korea; gpyo99@gmail.com(G.K.), seoyeopark42@gmail.com (S. P.)
* Correspondence: koo@inu.ac.kr and sungjun@dongguk.edu

Citation: . *Metals* **2024**, *8*, x. https://doi.org/10.3390/xxxxx

Academic Editor:

Received: date
Accepted: date
Published: date

Publisher's Note: MDPI stays neutral with regard to jurisdictional claims in published maps and institutional affiliations.

Copyright: © 2024 by the authors. Submitted for possible open access publication under the terms and conditions of the Creative Commons Attribution (CC BY) license (http://creativecommons.org/licenses/by/4.0/).

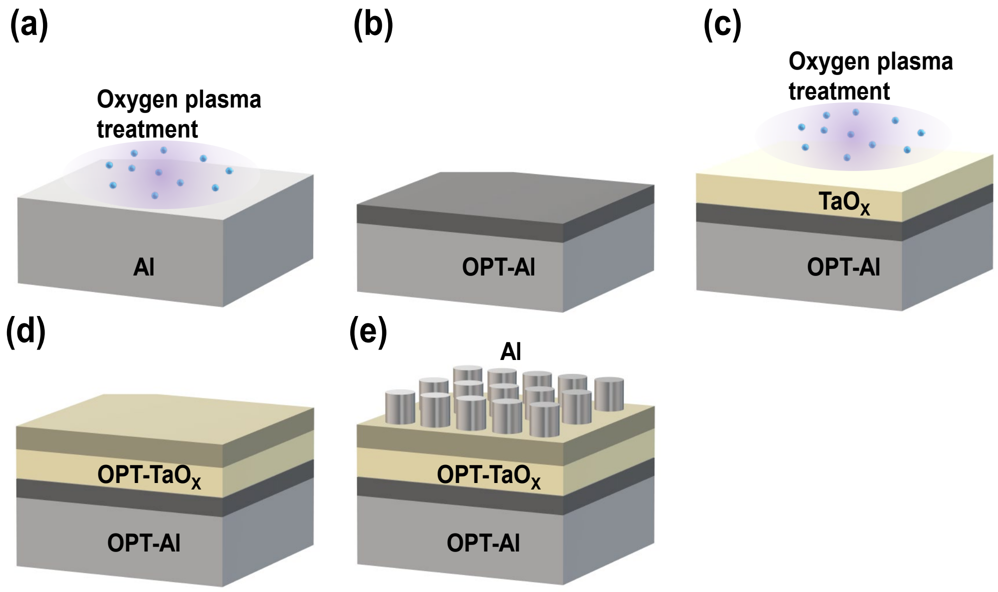

Figure S1. (a - e) Al/TaOx (OTP)/Al (OTP) device fabrication process schematic diagram.

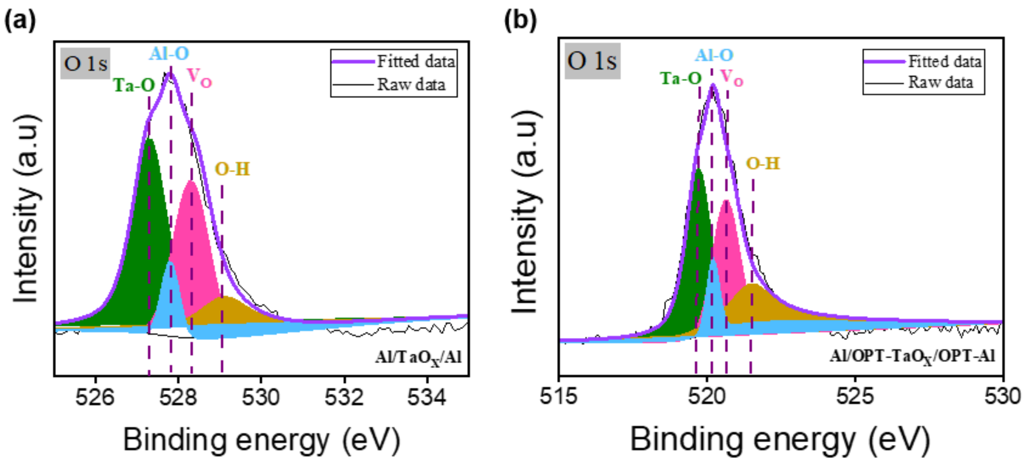

Figure S2. X-ray photoelectron spectra (XPS) of O 1s for (a) Al/TaOx/Al and (b) Al/TaOx (OPT)/Al (OPT).

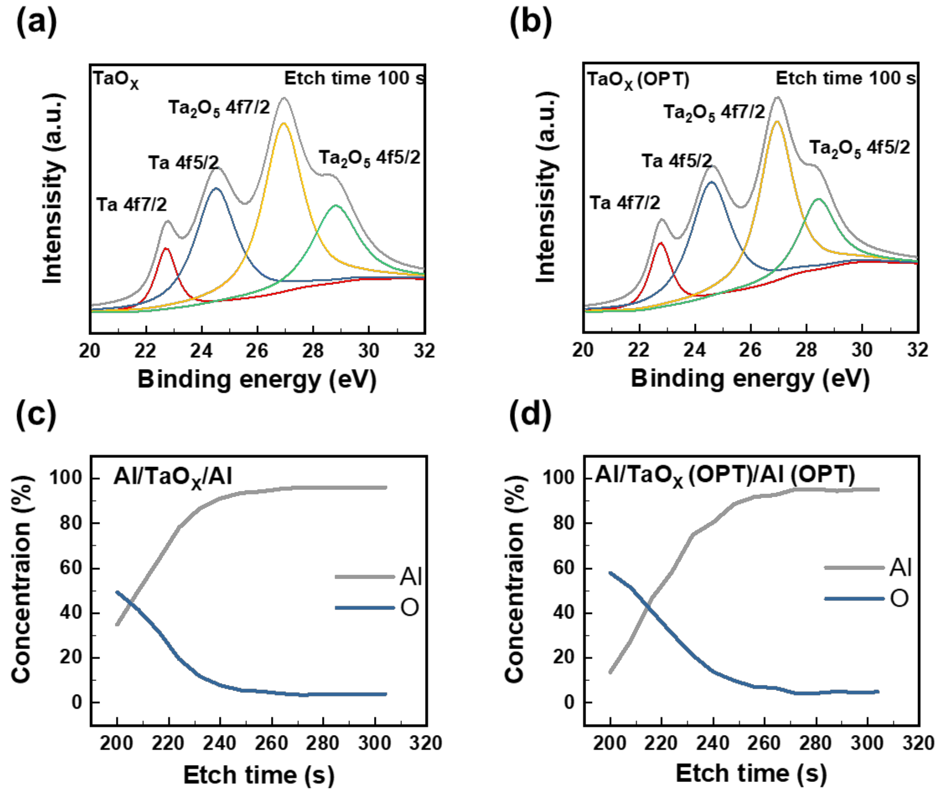

Figure S3. XPS spectra of Ta 4f on the (a) TaOx layer and (b) TaOx layer with O_2 plasma treatment when the etch time was 100 s. XPS depth profiles of Al and O elements in (c) Al/TaOx/Al device and (d) Al/TaOx (OPT)/Al (OPT) device.

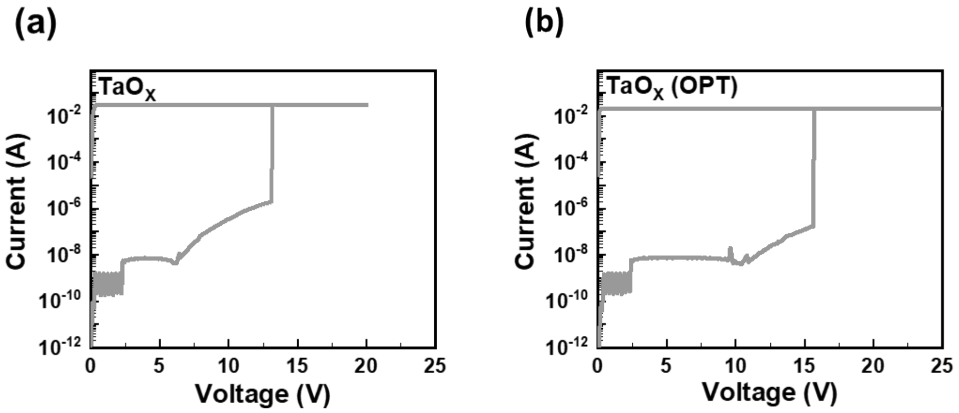

Figure S4. Forming process of the TaOx based RRAM each device (a) Al/TaOx/Al device and (b) Al/TaOx (OPT)/Al (OPT).

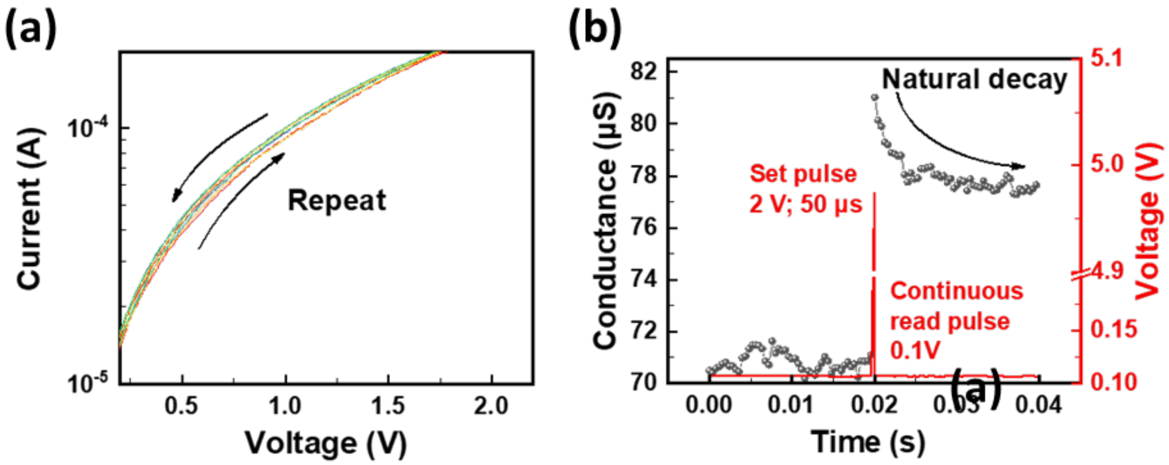

Figure S5. Partial short-term characteristics. (a) Set area in I-V curve: set process with a small window that repeats itself without a reset process. (b) Continuous read pulse and set plus. The conductance increases immediately after the set pulse and then naturally decay.

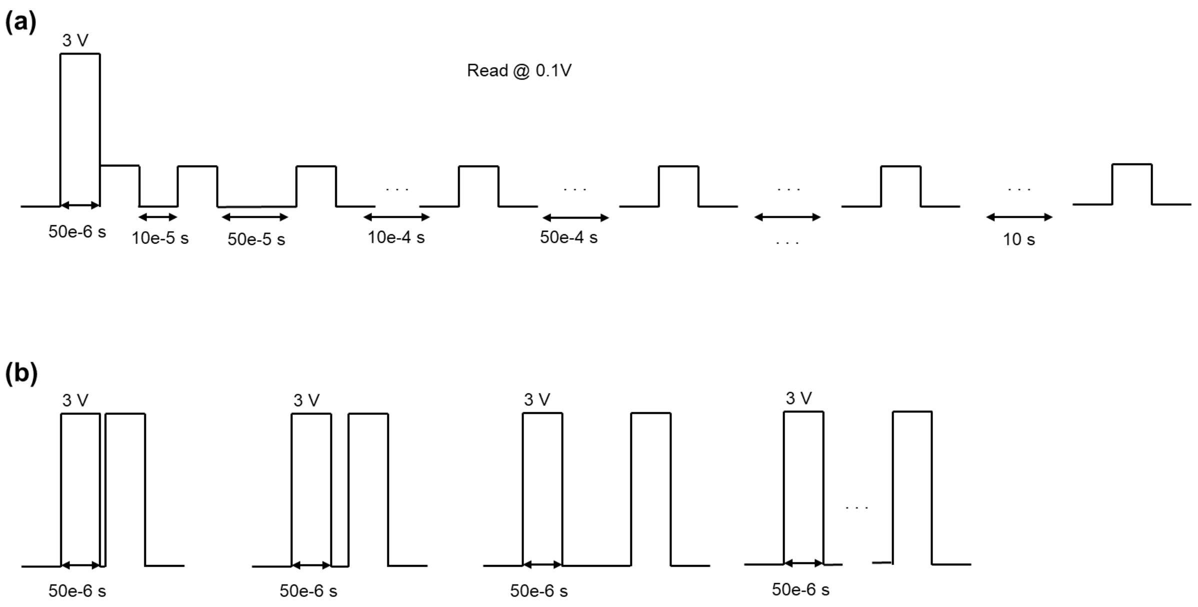

Figure S6. (a) Initial set pulses and read pulse with delay. (b) Pulse scheme of paired-pulse facilitation (PPF); With a delay between each two pulses (1 us, 10 us, 100 us, 1 ms).

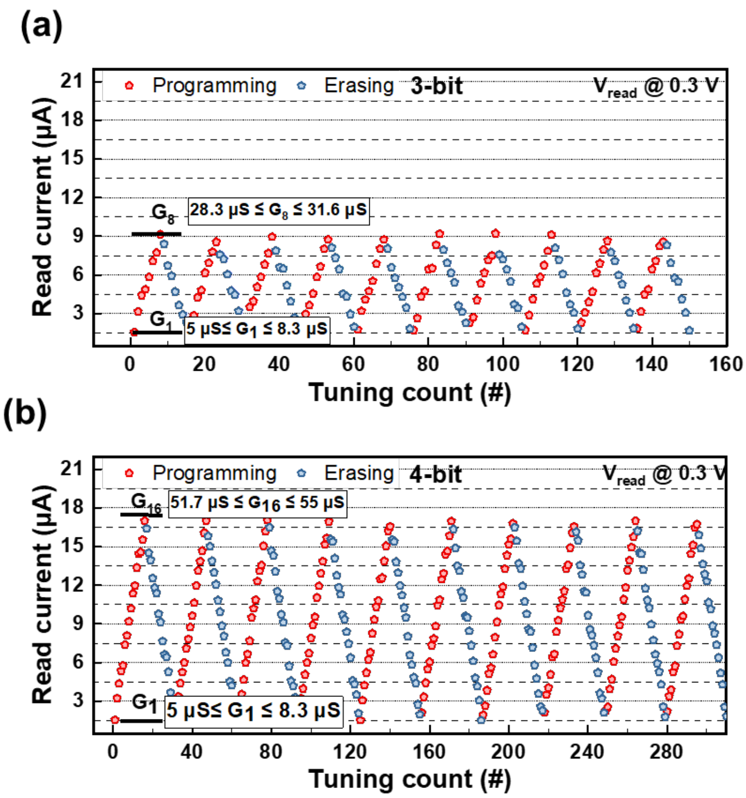

Figure S7. Multilevel switching characteristics of Al/TaOx (OPT)/A l(OPT) device over 10 cycles for (a) 3-bit MLC and (b) 4-bit MLC

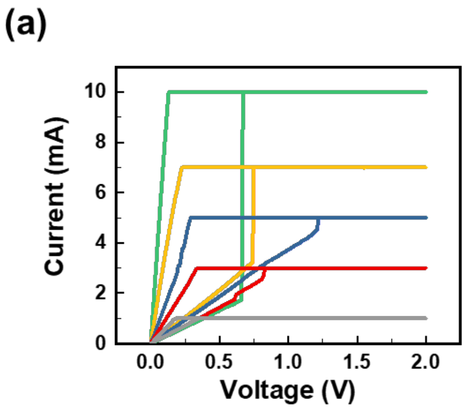

Figure S8. (a) DC I–V curves of the D2 with different CC conditions (1 mA, 3 mA, 5 mA, 7 mA, 10 mA).

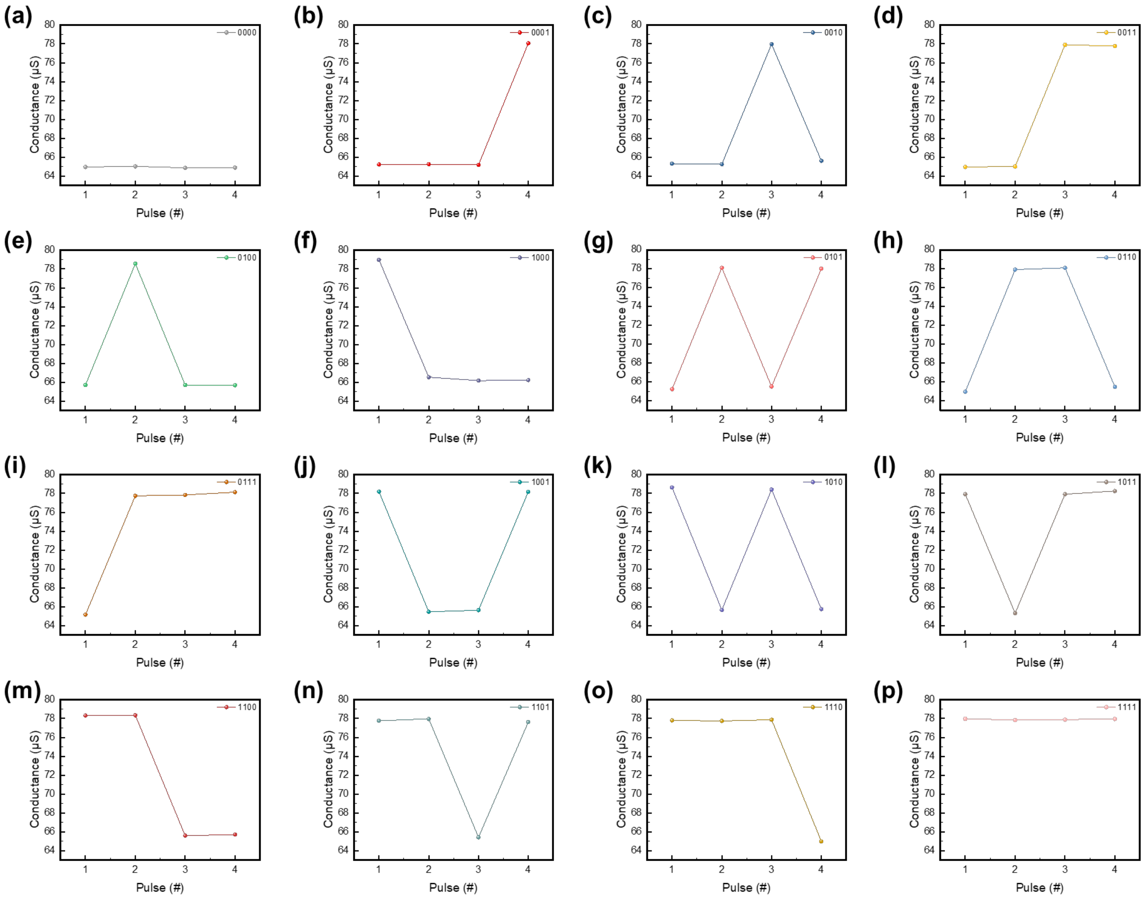

Figure S9. (a - p) Each sixteen states as per specific pulse streams.
